# CNS penetration of potential anti-COVID-19 drugs

**DOI:** 10.1007/s00415-020-09866-5

**Published:** 2020-05-02

**Authors:** Peter J. Richardson, Silvia Ottaviani, Alessandro Prelle, Justin Stebbing, Giacomo Casalini, Mario Corbellino

**Affiliations:** 1 BenevolentAI, London, UK; 2 Department of Surgery and Cancer, Imperial College, London, W12 0NN UK; 3 Department of Neurology and Stroke Unit, ASST Ovest Milanese, Milan, Italy; 4 III Division of Infectious Diseases, ASST Fatebenefratelli-Sacco, Milan, Italy

Dear Sirs,

In the context of the current COVID-19 pandemic, the ability of coronaviruses to enter the CNS and cause neurological deficits has been largely overlooked despite the fact that the closely related SARS virus was shown to be present in the CNS of SARS patients at autopsy [1]. The most likely CNS access routes include direct spread through the blood brain barrier or via the olfactory nerve, since intranasal infection of mice with either SARS or MERS results in virus access to the brain. It is, therefore, likely that SARS-CoV-2 can also penetrate the CNS. This would be facilitated by the expression of the SARS-CoV-2 receptor ACE2 in the brain, where it acts as a cell surface peptidase present on the surface of endothelial cells and neurons [2].

Consistent with this cases of encephalitis have been reported in patients with COVID-19, associated with either negative [3] or positive detection of SARS-CoV-2 in the CSF [4–6]. In a recent study of 214 hospitalised patients in Wuhan, 36.4% showed neurologic symptoms (headache, dizziness, impaired consciousness, ataxia, acute cerebrovascular disease, and epilepsy), with the more severely ill patients exhibiting cerebrovascular disease and epilepsy [7]. Similarly a study in Strasbourg described agitation and confusion with frontotemporal hypoperfusion [8]. Although it is clear that the pulmonary, renal, and cardiac damage are the primary causes of fatalities in COVID-19 patients, any cerebrovascular or neuronal damage that occurs during the disease could contribute. In addition, it is likely that virus-induced neurological damage could have consequences for surviving patients, with a dysexecutive syndrome being observed in up to one third of discharged patients [8].

There are a large number (as of 13th April, 179) of repurposing clinical trials testing drugs for COVID-19, so we have assessed the potential CNS penetration of the six most common drugs(Table 1). This assumes that brain penetration is largely similar between rodents, non-human primates and human patients, although it is possible that some of the poorly penetrating drugs could achieve higher concentrations if the blood brain barrier is compromised by the virus. The drug most likely to penetrate the brain is the anti-malarial hydroxychloroquine which, as of 13th April, was in 71 clinical trials, closely followed by the anti-rheumatoid JAK inhibitor baricitinib (Olumiant). In our previous correspondence we suggest that the combined anti-inflammatory and AI-predicted antiviral activities [9, 10] of the rheumatoid arthritis drug baricitinib would be potentially a effective treatment for those infected with SARS-CoV-2. This has been confirmed in further studies [11] where we reported that patients showed a reduction of symptoms (fever, cough) and a reduction in viral titre (nasopharyngeal swab and blood) and IL-6 on treatment with baricitinib for 10–12 days. Baricitinib is now being tested in randomised clinical trials including the large US NIAID study, as is another JAK inhibitor ruxolitinib, which has a lower brain penetrating potential. Of the remaining drugs being widely tested the lopinavir/ritonavir combination Kaletra (eight current trials) shows low brain penetrance. Tocilizumab, the anti-IL6R antibody (17 trials) shows a predictable low brain penetrance as does the modified nucleoside remdesivir (8 trials). Favipiravir, the RNA-dependent RNA polymerase inhibitor is being tested in eight trials, but shows low brain penetrance. Finally, the antibiotic azithromycin which is in 17 clinical trials shows good brain penetrance but negligible CSF concentrations, perhaps due to high-affinity brain tissue binding.

**Table 1:** Measured CNS exposures of potential COVID-19 therapeutics

	Brain: plasma ratio	Species	References
Hydroxychloroquine	21%	Mouse	[13]
Baricitinib	20%	Mouse	[12]
Ruxolitinib	3.5%	Mouse	[14]
Remdesivir	< 5%	Macaque	[15]
Tocilizumab	0.1%	Macaque	[16]
Lopinavir/ritonavir	0.02%	Human	[17]
Favipiravir^a^	Low	Mouse	[18]
Azithromycin^b^	260X	Human	[19]

^a^Active metabolite

^b^CSF extremely low

In our small series of patients dosed with baricitinib, one patient experienced severe ongoing visual hallucinations which resolved after the first few doses of baricitinib, perhaps suggesting that baricitinib can reduce neurological deficits arising from SARS-CoV-2 infection. Clearly, we were unable to determine the cause of these hallucinations but it is intriguing to us that the potent anti-inflammatory effect of baricitinib, acting centrally, peripherally or both was responsible. Since baricitinib has been shown to reduce the neurocognitive deficits associated with cerebral HIV-1 infection in mice [12], a direct anti-inflammatory action in the brain could be involved, especially given the brain exposures achieved with this drug. The CSF of two other COVID-19 patients in our clinics who showed encephalitis-type symptoms was tested for SARS-CoV-2 genetic material and proved to be negative, suggesting that such symptoms could also be a consequence of peripheral viral action or perhaps frontotemporal hypoperfusion.

In conclusion, some patients exhibiting neurological symptoms might have virus within the CNS whereas others do not. In this pandemic we should perhaps use well-tolerated brain penetrating drugs to ensure that any neurological consequences of SARS-CoV-2 infection are minimised.
